# Influence of self‐esteem on health‐related quality of life in children and adolescents with autism spectrum disorders

**DOI:** 10.1002/pcn5.70079

**Published:** 2025-03-12

**Authors:** Minobu Ikehara, Natsuko Kashida, Rio Ishida, Ryo Mizui, Manabu Makinodan, Kazuhiko Yamamuro

**Affiliations:** ^1^ Department of Psychiatry Nara Medical University School of Medicine Kashihara Nara Japan; ^2^ Department of Psychiatry Fujita Health University School of Medicine Toyoake Aichi Japan; ^3^ Division of Transformative Psychiatry and Synergistic Research International Center for Brain Science, Fujita Health University Toyoake Aichi Japan; ^4^ Center for Health Control, Nara Medical University Kashihara Nara Japan

**Keywords:** autism spectrum disorder, depressive symptoms, health‐related quality of life, parent–child discrepancies, self‐esteem

## Abstract

**Aim:**

Autism spectrum disorder (ASD) is a neurodevelopmental condition that markedly impairs the physical, emotional, and social domains of health‐related quality of life (HRQOL). Children with ASD typically report lower HRQOL than their neurotypical peers. This study investigated the impact of self‐esteem and depressive symptoms on HRQOL in children with ASD and explored the discrepancies between parent‐reported and self‐reported HRQOL.

**Methods:**

This study involved 94 participants, comprising 50 children with ASD and 44 typically developed. HRQOL was measured using the J‐KIDSCREEN‐52 (self‐reported and parent‐reported). Self‐esteem, depressive symptoms, and social support were assessed using the Rosenberg Self‐Esteem Scale, the Depression Self‐Rating Scale for Children, and the Multidimensional Scale of Perceived Social Support, respectively. Discrepancies between parent‐reported and self‐reported HRQOL were examined. Multiple regression analyses were performed to determine the influence of depressive symptoms and self‐esteem on HRQOL.

**Results:**

Children with ASD showed markedly lower HRQOL than their neurotypical peers. Discrepancies between parent‐reported and self‐reported HRQOL revealed differing perspectives. Higher depressive symptoms were strongly correlated with poorer HRQOL. Conversely, higher self‐esteem was linked to better HRQOL, notably in terms of self‐perception. Social support also markedly influenced HRQOL.

**Conclusion:**

This study underscores the necessity of addressing depressive symptoms, self‐esteem, and social support as interventions to enhance HRQOL in children with ASD. The differences between parent‐reported and self‐reported HRQOL highlight the need to incorporate both views into clinical assessments for comprehensive and effective interventions. Future research should explore these dynamics longitudinally and across diverse populations to refine the intervention strategies.

## Graphical abstract

This study examined the impact of self‐esteem and depressive symptoms on health‐related quality of life (HRQOL) in children with autism spectrum disorder, highlighting significant discrepancies between parent‐reported and self‐reported HRQOL. Higher depressive symptoms were strongly correlated with poorer HRQOL. Conversely, higher self‐esteem was linked to better HRQOL. Social support also markedly influenced HRQOL.

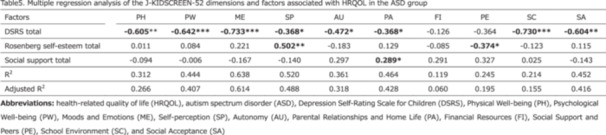

## INTRODUCTION

Autism spectrum disorder (ASD) is a pervasive neurodevelopmental condition characterized by notable difficulties in social communication, restricted interests, and repetitive behaviors.^1^ These core features often lead to profound challenges in everyday functioning, making it difficult for affected individuals to form meaningful social relationships, succeed in academic environments, and manage daily life tasks independently. Globally, ASD affects approximately 1.5% of children, with recent estimates in the United States indicating a prevalence of 1 in 54 among 8‐year‐olds.^2^ The rising prevalence of ASD has brought the condition to the forefront of public health concerns, particularly as the long‐term outcomes of these individuals depend heavily on early and effective intervention.^3^, ^4^, ^5^

One of the critical areas affected by ASD is health‐related quality of life (HRQOL), a multidimensional concept that encompasses an individual's physical, emotional, and social well‐being. Children and adolescents with ASD consistently report lower HRQOL compared to their neurotypical peers.^6^ This reduced quality of life can be attributed to the core symptoms of ASD, which often result in social isolation, difficulties in communication, and behavioral challenges. Moreover, many individuals with ASD experience comorbid conditions such as anxiety, depression, sleep disturbances, and gastrointestinal issues, which further exacerbate their overall quality of life.^7^ These factors combine to create a cycle of emotional and physical challenges that severely impact HRQOL across multiple dimensions.

A key aspect in evaluating HRQOL in children with ASD is the potential discrepancy between parent‐ and self‐reported outcomes. Parents may overestimate or underestimate their child's quality of life, particularly in domains related to emotional and social functioning.^6^, ^8^ These discrepancies can result from differences in perceptions between parents and children regarding the child's daily experiences and challenges. Understanding and addressing these gaps are essential for developing more accurate assessments and tailored interventions that consider both perspectives. Thus, this study explores parent–child discrepancies in HRQOL assessments, highlighting the importance of considering both viewpoints to gain a comprehensive understanding of children's well‐being.

Among the various factors that influence HRQOL in children and adolescents with ASD, self‐esteem has emerged as a critical yet underexplored variable. Self‐esteem, defined as an individual's perception of their own worth and abilities, plays a marked role in shaping psychological well‐being and social functioning. For children with ASD, low self‐esteem may intensify feelings of inadequacy, leading to further social withdrawal and increased vulnerability to mental health issues, such as anxiety and depression.^9^, ^10^ Despite its apparent importance, the relationship between self‐esteem and HRQOL in this population has not been thoroughly investigated. While the positive impact of self‐esteem on HRQOL has been reported in neurotypical children and in those with other psychiatric conditions, research specifically focusing on how self‐esteem affects HRQOL in children and adolescents with ASD is lacking.^8^, ^11^

The present study seeks to address this critical gap in the literature by examining the influence of self‐esteem on HRQOL in children and adolescents with ASD. We hypothesized that higher self‐esteem is positively associated with better HRQOL outcomes, including improved emotional well‐being, social functioning, and overall life satisfaction. By exploring these relationships, our study aimed to provide valuable insights that can inform the development of targeted interventions to enhance both the psychological and physical well‐being of young individuals with ASD. We believe that such interventions could play a crucial role in improving the long‐term outcomes in this growing population.

## METHODS

### Participants

We compared 50 participants (33 males and 17 females) aged 8–18 years diagnosed with ASD according to the Diagnostic and Statistical Manual of Mental Disorders Fifth Edition (DSM‐5)^12^ with 44 typically developing (TD) participants (29 males and 15 females) aged 8–18 years (Table 1). The participants with ASD had no history of treatment for psychiatric disorders and consulted an experienced psychiatrist at the Department of Psychiatry of Nara Medical University. The participants with ASD also underwent a standard clinical assessment comprising a psychiatric evaluation and Autism Diagnostic Observation schedule, 2nd Edition.^13^ Two experienced psychiatrists confirmed the ASD diagnosis according to the DSM‐5. Additionally, the cognitive function of each participant was measured using the Das‐Naglieri Cognitive Assessment System (DN‐CAS), a theory‐driven assessment kit based on the Planning, Attention, Simultaneous, and Successive (PASS) theory, which was utilized to assess cognitive function. PASS theory has reconceptualized intelligence as a process‐driven understanding of cognitive abilities based on the following four cognitive processes: planning, attention, simultaneous and successive processing,^14^ excluding individuals with scores below the total score of 70.

**Table 1:** Background.

	TD		ASD		
	Average	SE	Average	SE	P‐value
Age (years)	11.61	2.63	12.43	2.73	0.15
Sex (male/female)	29/15		33/17		0.993
DSRS	7.98	5.27	15.23	7.21	<0.001
Rosenberg self esteem	28.70	4.99	23.82	6.01	<0.001
MSPSS					
Social support total	67.64	13.15	55.10	16.60	0.001
Social support from family	22.94	4.36	19.51	6.47	0.004
Social support from significant other	22.59	4.91	19.90	6.23	0.002
Social support from friends	22.09	5.38	16.69	7.76	<0.001

Abbreviations: ASD, autism spectrum disorder; DSRS, Depression Self‐Rating Scale; MSPSS, multidimensional scale of perceived social support; SE, standard error; TD, typical developed.

Patients with comorbid psychiatric or neurological disorders, head injuries, serious medical conditions, or a history of substance abuse or dependence were excluded from the study. To ensure that the TD group had no psychiatric diagnoses, a standard clinical assessment was conducted, which included a psychiatric evaluation and the Japanese version of the Structured Clinical Interview for DSM‐IV Axis I Disorders/Non‐Patient Edition.^15^ TD participants were recruited through local print advertisements and underwent a comprehensive clinical assessment, including a psychiatric evaluation, structured diagnostic interviews, and a medical history review conducted by two experienced psychiatrists. A psychologist assessed the cognitive function of the participants using the DN‐CAS. The final TD group comprised 44 TD individuals without confirmed ASD and no current or history of psychiatric or neurological disorders were enrolled in the study. This study was approved by the Institutional Review Board of Nara Medical University. Written informed consent was obtained from each participating student aged 18 years or older. For students under 18 years of age, the purpose and nature of the study were explained to both the student and their parents, and written consent was obtained from one parent. This study was conducted in accordance with the principles of the Declaration of Helsinki.

### Japanese version of KIDSCREEN‐52

KIDSCREEN‐52 was developed as part of the KIDSCREEN project funded by the European Commission from 2001 to 2004. It was designed to assess children's perspectives on their health status.^16^, ^17^ The questionnaire consists of 52 items categorized into 10 dimensions (number of items including each dimension): physical well‐being (five items), psychological well‐being (six items), moods and emotions (seven items), self‐perception (five items), autonomy (five items), parental relationships and home life (six items), financial resources (three items), social support and peers (six items), school environment (six items), and social acceptance (three items). The participants were asked to respond based on their feelings and experiences during the past week. The KIDSCREEN‐52 uses a five‐point Likert scale to measure either the frequency of a behavior or feeling, or the intensity of an attitude. Negatively worded items were reverse‐scored and the total scores for each dimension were calculated. These scores are then transformed into *T*‐scores, with a mean of 50 and a standard deviation of 10, based on a representative sample of the European general population.^18^ A *T*‐score is not always an integer.

KIDSCREEN‐52 offers several advantages. Firstly, it is suitable for cross‐cultural use, as it was developed in multiple countries and tested in diverse populations. Secondly, it comprehensively covers the physical, psychological, social, and behavioral aspects of well‐being and functioning from a child's perspective. Thirdly, it provides both self‐reported and parent‐reported versions, with the parent version rewording the items from the child version. In this study, we utilized both the self‐reported and parent‐reported versions. The questionnaire takes approximately 10–20 min to complete and is intended for children and adolescents aged 8–18 years. Nezu et al. confirmed acceptable levels of reliability and validity of the Japanese version of KIDSCREEN‐52 (J‐KIDSCREEN‐52).^19^

### The Rosenberg Self‐Esteem Scale

The Rosenberg Self‐Esteem Scale (RSES) is a 10‐item measure developed by Rosenberg.^20^ For this study, we used the Japanese version translated by Yamamoto et al.^21^ The RSES evaluates self‐esteem, with participants rating statements like “I feel good enough” on a five‐point Likert scale. The total score ranges from 10 to 50, with higher scores indicating higher self‐esteem. Sakurai has confirmed the validity of the Japanese version of the RSES.^22^

### Depression Self‐Rating Scale for Children

The Depression Self‐Rating Scale (DSRS) is a psychological assessment tool used to evaluate depressive symptoms in individuals, particularly children and adolescents.^23^ The scale is designed as a self‐report measure in which respondents answer questions based on their own experiences and feelings. The DSRS is intended to provide an initial assessment of depression severity, helping identify individuals who may need further psychological evaluation or intervention. The DSRS typically includes a series of statements related to mood, behavior, and physical symptoms associated with depression. Respondents rated how often they experienced these symptoms over a specific time period, usually in the past week. The responses are usually given on a scale, such as “most of the time,” “sometimes,” or “never,” and the total score is calculated to gauge the level of depressive symptoms. This scale is commonly used in clinical settings, schools, and research studies. It is valued for its simplicity, which makes it easy to administer and interpret. However, the DSRS is not a diagnostic tool: it serves as a screening instrument that can highlight the need for a more comprehensive evaluation by mental health professionals.

### The Multidimensional Scale of Perceived Social Support

The Multidimensional Scale of Perceived Social Support (MSPSS) is a psychometric tool designed to assess perceived social support from three sources: family, friends, and significant others.^24^ The scale consists of 12 items, with four items dedicated to each source. The items assessing family support include: “My family really tries to help me,” “I get the emotional help and support I need from my family,” “I can talk about my problems with my family,” and “My family is willing to help me make decisions.” For friends, the items are: “My friends really try to help me,” “I can count on my friends when things go wrong,” “I have friends with whom I can share my joys and sorrows,” and “I can talk about my problems with my friends.” Regarding significant others, the items are: “There is a special person who is around when I am in need,” “There is a special person with whom I can share my joys and sorrows,” “I have a special person who is a real source of comfort to me,” and “There is a special person in my life who cares about my feelings.” Each item is rated on a seven‐point Likert scale ranging from 1 (very strongly disagree) to 7 (very strongly agree), with higher scores indicating greater perceived support. The MSPSS is widely recognized for its reliability and validity, making it a valuable tool in both research and clinical applications.^25^

### Statistical analyses

All statistical analyses were conducted using SPSS 24.0 (IBM Corporation). Differences in mean *T*‐scores between the ASD and TD groups, as well as between self‐reported and parent‐reported scores within each dimension of the J‐KIDSCREEN‐52 were tested using Student's *t*‐test. We applied Bonferroni correction to adjust for multiple comparisons. The internal consistency of each dimension of the J‐KIDSCREEN‐52, RSES, and MSPSS was evaluated using Cronbach's *α* coefficients. Test–retest reliability was assessed by examining the intraclass correlation coefficient (ICC) between self‐reported and parent‐reported scores using a one‐way random effects model, with a coefficient of ≥0.60 considered indicative of adequate stability.^26^ Statistical significance was set at P < 0.05. Multiple regression analyses were performed with J‐KIDSCREEN‐52 dimension scores of self‐reported as dependent variables and DSRS and RSES as independent variables. A stepwise method was used for variable selection, and an adjusted *R*² was used to assess the goodness of fit. Analyses using J‐KIDSCREEN‐52 scores were basically carried out using *T*‐scores, but raw data before transformation into *T*‐scores were used for analyses of discrepancies between self‐reported and parent‐reported scores and ICC.

## RESULTS

### Demographic and clinical data

A total of 94 participants were included in the study, with 50 children in the ASD group and 44 children in the TD group. Demographic and clinical characteristics of the participants are shown in Table 1. The mean age of the ASD group was 12.43 years (SD = 2.73), compared to 11.61 years (SD = 2.63) in the TD group. This age difference was not statistically significant (P = 0.15). The sex distribution was nearly identical, with 33 males and 17 females in the ASD group and 29 males and 15 females in the TD group (P = 0.993), indicating no significant difference in sex composition.

In terms of psychological and social characteristics, the ASD group exhibited significantly higher DSRS scores, with a mean score of 15.23 (SD = 7.21) compared to 7.98 (SD = 5.27) in the TD group (P < 0.001). This finding highlights the greater prevalence of depressive symptoms among children with ASD. Additionally, self‐esteem, as measured by the RSES, was significantly lower in the ASD group (23.82 ± 6.01) than in the TD group (28.70 ± 4.99, P < 0.001), suggesting lower self‐worth in children with ASD.

Social support, as measured by MSPSS, was also significantly reduced in the ASD group. The total social support score was 55.10 (SD = 16.60) in the ASD group, compared to 67.64 (SD = 13.15) in the TD group (P = 0.001). Specific domains of social support, including family (19.51 ± 6.47 vs. 22.94 ± 4.36, P = 0.004), significant others (19.90 ± 6.23 vs. 22.59 ± 4.91, P = 0.002), and friends (16.69 ± 7.76 vs. 22.09 ± 5.38, P < 0.001), were all significantly lower in the ASD group. These data reflect the diminished social networks and support systems available to children with ASD.

### Differences in self‐reported J‐KIDSCREEN‐52 between ASD and TD

The study also revealed significant differences in HRQOL between the ASD and TD groups, as measured by J‐KIDSCREEN‐52 (Table 2). Self‐reported scores indicated that children with ASD experienced significantly lower HRQOL in multiple dimensions than their neurotypical peers. Specifically, the ASD group had lower scores in physical well‐being (44.88 ± 11.91 vs. 58.14 ± 11.96, P < 0.001), psychological well‐being (40.79 ± 10.95 vs. 52.20 ± 11.77, P < 0.001), moods and emotions (43.98 ± 12.28 vs. 54.09 ± 10.54, P < 0.001), autonomy (45.84 ± 8.50 vs. 52.71 ± 10.74, P = 0.008), parental relationships and home life (44.80 ± 10.17 vs. 51.92 ± 10.77, P = 0.014), social support and peers (39.66 ± 13.90 vs. 53.49 ± 14.13, P < 0.001), and school environment (44.59 ± 13.78 vs. 56.13 ± 12.25, P < 0.001). These findings indicate that children with ASD perceived their physical and psychological health to be poorer than those in the TD group.

**Table 2:** Self report.

J‐KIDSCREEN‐52	TD			ASD			95% CI		P‐value
	Mean	SD	Internal consistency	Mean	SD	Internal consistency			
Dimensions			Cronbach's *α*			Cronbach's *α*			
Physical well‐being	58.14	11.96	0.69	44.88	11.91	0.83	8.37	18.16	<0.001
Psychological well‐being	52.20	11.77	0.94	40.79	10.95	0.90	6.76	16.07	<0.001
Moods and emotions	54.09	10.54	0.85	43.98	12.28	0.89	5.40	14.84	<0.001
Self‐perception	47.86	7.89	0.50	44.15	9.46	0.67	0.12	7.31	0.431
Autonomy	52.71	10.74	0.83	45.84	8.50	0.68	2.85	10.88	0.010
Parental relationships and home life	51.92	10.77	0.91	44.80	10.17	0.89	2.83	11.41	0.014
Financial resources	48.56	11.22	0.89	44.88	12.14	0.89	−1.13	8.49	1.000
Social support and peers	53.49	14.13	0.92	39.66	13.90	0.91	8.08	19.58	<0.001
School environments	56.13	12.25	0.94	44.59	13.78	0.90	6.17	16.91	<0.001
Social acceptance	52.19	9.22	0.77	46.41	11.50	0.64	1.54	10.03	0.082

*Note*: T‐scores were used to analyze each J‐KIDSCREEN‐52 dimension.

Abbreviations: ASD, autism spectrum disorder; SD, standard deviation; TD, typical developed.

### Differences in parent‐reported J‐KIDSCREEN‐52 between ASD and TD

In addition to self‐reported outcomes, parent‐reported J‐KIDSCREEN‐52 scores further support these findings (Table 3). The ASD group scored significantly lower in physical well‐being (46.88 ± 14.70 vs. 60.72 ± 10.65, P < 0.001), psychological well‐being (39.80 ± 12.20 vs. 49.64 ± 10.75, P < 0.001), moods and emotions (42.95 ± 10.72 vs. 53.97 ± 10.13, P < 0.001), parental relationships and home life (39.90 ± 8.80 vs. 45.79 ± 10.36, P = 0.037), social support and peers (36.80 ± 17.52 vs. 50.34 ± 15.46, P = 0.002), and school environment (42.06 ± 17.39 vs. 51.43 ± 11.17, P = 0.029). These findings indicate the potential challenges in the family dynamics of children with ASD.

**Table 3:** Observer.

J‐KIDSCREEN‐52	TD			ASD			95% CI		P‐value
	Mean	SD	Internal consistency	Mean	SD	Internal consistency			
Dimensions			Cronbach's *α*			Cronbach's *α*			
Physical well‐being	60.72	10.65	0.75	46.88	14.70	0.89	8.63	19.07	<0.001
Psychological well‐being	49.64	10.75	0.93	39.80	12.20	0.95	5.10	14.58	<0.001
Moods and emotions	53.97	10.13	0.81	42.95	10.72	0.89	6.73	15.31	<0.001
Self‐perception	47.44	8.84	0.59	43.89	10.04	0.80	−0.34	7.46	0.734
Autonomy	50.09	9.72	0.72	46.41	10.74	0.79	−0.54	7.90	0.865
Parental relationships and home life	45.79	10.36	0.90	39.90	8.80	0.86	1.96	9.81	0.037
Financial resources	50.51	12.04	0.97	48.95	12.40	0.87	−3.46	6.58	1.000
Social support and peers	50.34	15.46	0.96	36.80	17.52	0.96	6.72	20.34	0.002
School environments	51.43	11.17	0.91	42.06	17.39	0.95	3.45	15.30	0.023
Social acceptance	52.15	8.61	0.64	49.07	10.80	0.85	−0.96	7.11	1.000

*Note*: T‐scores were used to analyze each J‐KIDSCREEN‐52 dimension.

Abbreviations: ASD, autism spectrum disorder; SD, standard deviation; TD, typical developed.

### Discrepancies between parent‐reported and self‐reported HRQOL scores

We calculated Δ values to assess discrepancies between parent‐reported and self‐reported HRQOL scores and evaluated them utilizing a paired *T*‐test. To calculate the Δ values, we initially determined the difference between the self‐reported and parent‐reported scores for each item for each participant and subsequently calculated the mean of these differences. Specifically, the Δ value is defined as the mean of the differences obtained by subtracting each participant's parent‐reported score from their self‐reported score. A positive Δ indicates a higher level of HRQOL reported by the child, while a negative Δ indicates a lower level of HRQOL reported by the child.

$$\Delta =\frac{\sum \;(\text{self}‐\text{reported score}-\text{parent}‐\text{reported score})}{\text{mumber of participants in the group}}.$$

Bonferroni correction adjusted for multiple comparisons, with significance set at a corrected P‐value threshold. ICC assessed concordance between parent and child ratings for each dimension.

Significant discrepancies in Δ values were found in the physical well‐being (Δ = −1.14, P < 0.05, Bonferroni corrected) and parental relationships and home life (Δ = 2.15, P < 0.01, Bonferroni corrected) dimensions among all participants. Parents overestimated their child's physical well‐being while underestimating family dynamics quality compared to their children's self‐reports. However, after Bonferroni correction, no significant differences in Δ values were observed across any dimension within the TD or ASD subgroups, suggesting no systematic bias between parent‐reported and self‐reported scores in these subgroups.

Despite these findings, ICC values showed considerable variability in parent–child agreement. Dimensions with ICC < 0.4 (bold in Table 4) indicated low agreement. In the TD group, low ICC values appeared in physical well‐being (ICC = 0.238), psychological well‐being (ICC = 0.155), moods and emotions (ICC = 0.133), self‐perception (ICC = 0.058), autonomy (ICC = 0.220), parental relationships and home life (ICC = 0.020), social support and peers (ICC = 0.329), and social acceptance (ICC = 0.224), highlighting inconsistency in parent–child agreement, especially in physical, emotional, and relational domains. Conversely, the ASD group showed low ICC values in moods and emotions (ICC = 0.281), parental relationships and home life (ICC = 0.362), and autonomy (ICC = 0.220). Thus, Δ values indicated no significant differences in the TD and ASD groups individually, but ICC values revealed perceptual discrepancies in both groups, with the TD group showing a wider range of dimensions with low parent–child agreement.

**Table 4:** Discrepancies between parent‐reported and self‐reported HRQOL.

J‐KIDSCREEN‐52 Dimensions	Δ (all participants)	Δ (TD)	ICC (TD)	Δ (ASD)	ICC (ASD)
Physical well‐being	−1.14*	−1.25	**0.238**	−1.04	0.719
Psychological well‐being	0.49	−1.09	**0.155**	−0.04	0.603
Moods and emotions	−1.22	−0.46	**0.133**	−1.90	**0.281**
Self‐perception	−0.70	−0.55	**0.058**	−0.84	0.514
Autonomy	−0.20	0.39	**0.220**	−0.72	**0.220**
Parental relationships and home life	2.15**	2.52	**0.020**	1.82	**0.362**
Financial resources	−0.47	−0.02	0.479	−0.86	0.448
Social support and peers	1.93	2.30	**0.329**	1.60	0.532
School environments	0.49	1.27	0.407	−0.20	0.518
Social acceptance	−0.29	0.05	**0.224**	−0.58	**0.331**

*Note*: Δ value is defined as the mean of the differences obtained by subtracting each participant's parent‐reported score from their self‐reported score. A positive Δ indicate a higher level of HRQOL reported by a child. A negative Δ indicate a lower level of HRQOL reported by a child. In calculating the Δ value and ICC for each J‐KIDSCREEN‐52 item, the raw scores before transformed into *T*‐scores were used. Values in bold indicate an ICC < 0.40.

Abbreviations: ASD, autism spectrum disorder; HRQOL, health‐related quality of life; ICC, intraclass correlation coefficient; TD, typical developed.

* P < 0.05.

### Multiple regression analyses in ASD

Multiple regression analyses highlighted the influence of self‐esteem, depressive symptoms, and social support on HRQOL (Table 5). In the ASD group, higher DSRS scores were associated with significantly lower HRQOL in terms of physical well‐being (*β* = −0.605, P < 0.01), psychological well‐being (*β* = −0.642, P < 0.001), moods and emotions (*β* = −0.733, P < 0.001), self‐perception (*β* = −0.368, P < 0.05), autonomy (*β* = −0.472, P < 0.05), parental relationships and home life (*β* = −0.368, P < 0.05), school environment (*β* = −0.730, P < 0.001) and social acceptance (*β* = −0.604, P < 0.01). These results suggest that depressive symptoms play an important role in reducing quality of children with ASD. Self‐esteem has also emerged as a significant factor affecting HRQOL outcomes. In the ASD group, higher self‐esteem was associated with better scores on the self‐perception dimension *(β* = 0.502, P < 0.01) but with lower scores on the social support and peers dimension (*β* = −0.374, P < 0.05). Social support, as assessed using the MSPSS, is another important determinant of HRQOL. In the ASD group, higher social support was linked to better parental relationships and home life scores (*β* = 0.289, P < 0.05).

**Table 5:** Multiple regression analysis of the J‐KIDSCREEN‐52 dimensions and factors associated with HRQOL in the ASD group.

Factors	PH	PW	ME	SP	AU	PA	FI	PE	SC	SA
DSRS total	−0.605**	−0.642***	−0.733***	−0.368*	−0.472*	−0.368*	−0.126	−0.364	−0.730***	−0.604**
Rosenberg self‐esteem total	0.011	0.084	0.221	0.502**	−0.183	0.129	−0.085	−0.374*	−0.123	0.115
Social support total	−0.094	−0.006	−0.167	−0.140	0.297	0.289*	0.291	0.327	0.025	−0.143
*R* ^2^	0.312	0.444	0.638	0.520	0.361	0.464	0.119	0.245	0.214	0.452
Adjusted *R* ^2^	0.266	0.407	0.614	0.488	0.318	0.428	0.060	0.195	0.155	0.416

Abbreviations: ASD, autism spectrum disorder; AU, autonomy; DSRS, Depression Self‐Rating Scale for Children; FI, financial resources; HRQOL, health‐related quality of life; ME, moods and emotions; PA, parental relationships and home life; PE, social support and peers; PH, physical well‐being; PW, psychological well‐being; SA, social acceptance; SC, school environment; SP, self‐perception.

These findings highlight the complex interplay between depressive symptoms, self‐esteem, and social support in determining HRQOL outcomes in children with ASD. Addressing these factors through targeted interventions could be the key to improving the quality of life of this population.

### Multiple regression analyses in TD

Multiple regression analysis revealed effects on HRQOL of self‐esteem, depressive symptoms, and social support in the TD group as well (Table 6). In the TD group, higher DSRS scores were associated with significantly lower HRQOL in terms of physical well‐being (*β* = −0.533, P < 0.05), psychological well‐being (*β* = −0.577, P < 0.01), moods and emotions (*β* = −0.649, P < 0.01), autonomy (*β* = −0.470, P < 0.05), and parental relationships and home life (*β* = −0.589, P < 0.01). In the TD group, higher self‐esteem was associated with improved scores on the social support and peers dimension (*β* = 0.445, P < 0.01), and social support influenced the dimension of school environment (*β* = 0.407, P < 0.05).

**Table 6:** Multiple regression analysis of the J‐KIDSCREEN‐52 dimensions and factors associated with HRQOL in the TD group.

Factors	PH	PW	ME	SP	AU	PA	FI	PE	SC	SA
DSRS total	−0.533*	−0.577**	−0.649**	−0.144	−0.470*	−0.589**	−0.411	0.015	−0.195	−0.376
Rosenberg self‐esteem total	0.184	−0.067	0.053	0.330	0.162	−0.101	−0.093	0.445**	0.072	0.093
Social support total	−0.127	0.265	0.005	0.121	−0.039	0.215	−0.126	0.337	0.407*	0.080
*R* ^2^	0.340	0.554	0.470	0.263	0.331	0.484	0.087	0.443	0.362	0.247
Adjusted *R* ^2^	0.290	0.520	0.431	0.207	0.259	0.446	0.018	0.390	0.314	0.191

Abbreviations: AU, autonomy; DSRS, Depression Self‐Rating Scale for Children; FI, financial resources; HRQOL, health‐related quality of life; ME, moods and emotions; PA, parental relationships and home life; PE, social support and peers; PH, physical well‐being; PW, psychological well‐being; SA, social acceptance; SC, school environment; SP, self‐perception; TD, typical developed.

## DISCUSSION

This study explored the HRQOL of children and adolescents with ASD and examined the influence of self‐esteem, depressive symptoms, and social support on HRQOL. Our findings confirm that children with ASD experience significantly lower HRQOL across multiple dimensions than their neurotypical peers. These results align with existing literature, which emphasizes the substantial challenges faced by individuals with ASD, particularly in areas such as physical well‐being, psychological health, and social functioning.^6^, ^7^, ^27^, ^28^

This study found significant discrepancies between parent‐reported and self‐reported HRQOL, especially in the physical well‐being and parental relationships and home life domains. These differences highlight the complex family dynamics and the difficulties parents face in accurately perceiving their child's well‐being. Previous research suggests parents typically underestimate their child's HRQOL,^29^, ^30^, ^31^ particularly in emotional and social areas, due to their less observable and subjective nature. However, this study observed both underestimations in parental relationships and home life and overestimation in physical well‐being, illustrating the multifaceted nature of these discrepancies.

Research has identified various sociodemographic and contextual factors affecting these discrepancies, such as the child's education level, parental education, and environmental stressors like noise or lack of outdoor spaces, which are linked to parental overestimation of HRQOL.^30^ Another study found that discrepancies in HRQOL reporting were more significant for subjective dimensions (e.g., emotional well‐being) than observable ones (e.g., physical well‐being).^29^ Researchers noted that agreement levels decreased with the child's age. These findings align with our study's observed overestimation of physical well‐being, likely due to parents' limited awareness of their children's subtle physical challenges in daily activities.

The underestimation in the parental relationships and home life domain may result from parental modesty or critical self‐evaluation. Studies show that reduced parental well‐being and high stress levels can contribute to this underestimation.^29^, ^30^ Additionally, parents' proxy ratings are affected by their mental health and socio‐environmental stressors, highlighting the importance of contextual factors in interpreting parent‐reported HRQOL scores.

Our findings showed that discrepancies were more pronounced in the TD group than in the ASD group across a broader range of HRQOL dimensions, as evidenced by ICC values. Although mean differences in parent‐reported and self‐reported HRQOL (Δ values) were not statistically significant in either group after Bonferroni correction, ICC values indicated substantial variability in individual‐level agreement. In the TD group, ICC < 0.4 was observed in many dimensions, including physical well‐being, psychological well‐being, moods and emotions, self‐perception, autonomy, parental relationships and home life, social support and peers, and social acceptance. In contrast, the ASD group showed ICC < 0.4 in fewer dimensions, such as moods and emotions, parental relationships and home life, and autonomy. This suggests that perceptual discrepancies in the TD group were more widespread, while in the ASD group, they were concentrated in specific areas.

The discrepancy may stem from the unique nature of parent–child interactions in families with children with ASD. Parents of children with ASD often spend more time directly engaging with their child due to frequent medical appointments, therapeutic interventions, and specialized education from an early age. These activities likely increase parental observation and communication, allowing better understanding of the child's challenges. This increased involvement may align parent‐reported and self‐reported HRQOL evaluations, especially in areas like physical well‐being, psychological well‐being, self‐perception, and social support and peers, where perceptual differences were less pronounced in the ASD group compared to the TD group. Conversely, parents of TD children might have fewer structured opportunities for direct communication about the child's subjective experiences, explaining the broader perceptual disparities in the TD group.

These findings highlight the importance of contextualizing parent–child interactions when interpreting HRQOL data. The improved alignment between parent‐reported and self‐reported evaluations in the ASD group suggests that interventions to enhance communication and mutual understanding between parents and children could also benefit the TD population. Facilitating open discussions about emotional well‐being and daily autonomy may reduce perceptual discrepancies in TD families. Future research should examine how specific parental behaviors, communication styles, and family dynamics affect parent–child concordance on HRQOL across various populations, focusing on tailoring interventions to meet each group's distinct needs.

One of the most striking findings of this study was the significant impact of depressive symptoms on HRQOL in children with ASD. The DSRS results showed that higher levels of depressive symptoms were associated with lower HRQOL, especially in the domains of physical well‐being, psychological well‐being, and moods and emotions. This is consistent with previous research indicating the high prevalence of depression in children with ASD and its exacerbating effect on the difficulties they face.^32^, ^33^, ^34^, ^35^ Given that depressive symptoms are treatable, this finding highlights the importance of early identification and treatment of depression in this population. Interventions such as cognitive behavioral therapies have shown promise in reducing depressive symptoms in children with ASD, thereby potentially improving their overall quality of life.^36^, ^37^, ^38^, ^39^

Self‐esteem significantly influences HRQOL, with children possessing higher self‐esteem reporting better outcomes in self‐perception and parental relationships and home life. This aligns with studies indicating self‐esteem's crucial role in emotional regulation and social interaction, which are particularly challenging for individuals with ASD.^40^, ^41^ The strong link between self‐esteem and HRQOL highlights the importance of interventions targeting self‐esteem, such as social skills training and supportive counseling, to improve the well‐being of children with ASD.

Social support, assessed by MSPSS, also predicts HRQOL. Children with ASD who report lower social support experience poorer HRQOL, especially in parental relationships and home life. This underscores social support's protective role in mitigating ASD's negative impact on quality of life. Strong social networks, including family, peers, and community support, are vital for helping children with ASD manage daily challenges.^42^, ^43^, ^44^, ^45^, ^46^, ^47^ Thus, creating supportive environments should be a primary focus of interventions to enhance their quality of life.

This study aligns with previous research highlighting the multidimensional challenges faced by children with ASD. Kuhlthau et al. found that children with ASD report lower HRQOL than those with other chronic conditions, especially in social functioning and emotional well‐being.^48^ Our results extend this understanding by demonstrating the significant impact of self‐esteem and depressive symptoms on HRQOL in this population. The influence of social support concurs with Weiss et al., who identified it as a crucial buffer against stress and mental health issues in children with ASD.^49^ Differences from previous studies were noted, while behavioral interventions are often emphasized for improving HRQOL, our findings suggest that psychological factors, such as self‐esteem and depression, may be equally or more important.^50^, ^51^, ^52^, ^53^, ^54^ This indicates the need for an integrated approach combining behavioral therapies with psychological support to address the comprehensive challenges faced by children with ASD.

This study has several limitations. The cross‐sectional design restricts causal inferences between self‐esteem, depressive symptoms, and HRQOL, necessitating longitudinal studies to examine these interactions over time and the impact of interventions on HRQOL. Moreover, the small sample size, especially in the ASD group, may limit the generalizability of findings, requiring future research to use larger, more diverse samples to verify results across different ASD subtypes and contexts. Additionally, reliance on self‐reported and parent‐reported measures may introduce bias, particularly in areas like self‐esteem and social support. Objective measures and qualitative data are needed to better understand the lived experiences of children with ASD.

## CONCLUSION

This study examined the effects of self‐esteem, depressive symptoms, and social support on the HRQOL of children and adolescents with ASD. Results show that children with ASD have significantly lower HRQOL across multiple dimensions compared to neurotypical peers. Self‐esteem, depressive symptoms, and social support significantly influence HRQOL, with higher depressive symptoms correlating with poorer HRQOL and higher self‐esteem linked to better self‐perception. Social support notably moderated HRQOL, highlighting the necessity of supportive environments for children with ASD.

These findings underscore the need for early mental health interventions targeting depressive symptoms and programs enhancing self‐esteem and social support in children with ASD to improve their quality of life. Future research should focus on longitudinal studies to understand the causal relationships between self‐esteem, depressive symptoms, social support, and HRQOL in ASD, and include diverse subgroups within the ASD population for more nuanced intervention strategies.

## AUTHOR CONTRIBUTIONS

Minobu Ikehara, Natsuko Kashida, Rio Ishida, Ryo Mizui, Manabu Makinodan, and Kazuhiko Yamamuro were involved in data collection. Minobu Ikehara and Kazuhiko Yamamuro wrote the manuscript. Kazuhiko Yamamuro supervised this study. All authors have read and approved the final version of the manuscript.

## CONFLICT OF INTEREST STATEMENT

The authors declare no conflicts of interest.

## ETHICS APPROVAL STATEMENT

This study was approved by the Institutional Review Board of Nara Medical University.

## PATIENT CONSENT STATEMENT

This study was approved by the Institutional Review Board of Nara Medical University, and written consent for participation was obtained from all participants.

## CLINICAL TRIAL REGISTRATION

This study was not conducted as a clinical trial, therefore this section is not applicable.

## Data Availability

Data are available from the corresponding author upon request.
